# Optimum pyrolysis conditions to prepare the most crystalline boron carbide powder from boric acid–mannitol complex ester

**DOI:** 10.55730/1300-0527.3620

**Published:** 2023-10-12

**Authors:** Abdullah Devrim PEKDEMİR, Müşerref ÖNAL, Yüksel SARIKAYA

**Affiliations:** 1 General Directorate of Mineral Research and Exploration, Ankara, Turkiye; 2 Department of Chemistry, Faculty of Science, Ankara University, Ankara, Turkiye

**Keywords:** Boric acid, boron carbide, ester, mannitol, pyrolysis

## Abstract

A weak acidic complex ester (CE) in solid form was prepared by a condensation reaction between very weak boric acid (BA: H_3_BO_3_) and (D)-mannitol (MA: C_6_H_14_O_6_) by the molar ratio of BA/MA = 2. A boron carbide (B_4_C) precursor was obtained from heating of the CE at 400 °C for 4 h. The precursor was pyrolyzed under argon flow in the interval of 1300–1550 °C for 4 h and at 1400 °C for 1–4 h, respectively. The materials were examined using several techniques such as X-ray diffraction analysis, thermal analysis, scanning electron microscopy, particle size distribution, and nitrogen adsorption/desorption. The optimum pyrolysis temperature and duration were 1400 °C and 4 h, respectively. The most crystalline B_4_C particles were distributed between 1 and 100 μm with a mean particle size of 20 μm. The specific surface area and specific pore volume were 13.5 m^2^ g^−1^ and 0.09 cm^3^ g^−1^, respectively. The size of the pores was between 2 and 36 nm with a mean size of 14 nm.

## 1. Introduction

According to previous experimental studies, boron carbide (B_4_C) includes many phases with different chemical composition, crystal structure, and morphology as well as their complex mixtures [1,2]. The idealized B_4_C, which has a rhombohedral crystal structure, is an almost covalently bonded compound having a 6% ionic character. Some exceptional properties of B_4_C are chemical inertness and low density (2.52 g mL^−1^) and thermal expansion coefficient (5.5 × 10^−6^ K^−1^) as well as high melting temperature (2450 °C), hardness (29.1 GPa), mechanical strength (350 MPa), elastic modulus (448 GPa), Seebeck coefficient (300 μV K^−1^), and cross section (600 barns = 6 × 10^−22^ cm^2^) for neutron absorption [3–6]. Two important disadvantages of the structure of B_4_C are low fracture toughness and poor sinterability at nearly 2000 °C [7].

Due to being the third hardest material after diamond and cubic boron nitride (BN), B_4_C has been used as a nonoxide ceramic for the cutting and polishing of metals and other softer ceramics [8]. Various machine parts, light mass armors for body and vehicle, protective coating of electronic surfaces, p-type semiconductors, thermocouples, diode and transistor devices, and control rods and shielding for nuclear reactors as well as materials for cancer therapy by neutron capturing have been manufactured using boron carbide and its composites [9–13]. Due to these various applications in our daily lives, various physicochemical properties have long been investigated [14–17] with ongoing detailed studies on B_4_C and its composite materials [18–25].

Synthesis methods, crystal structure, and sintering of boron carbide were extensively studied [3,26]. It can be synthesized from its elements at high temperatures and pressures [27]. The methodologies used to obtain fine B_4_C powder at lower temperature are safe, beneficial, and more economical than those used at higher temperature, which almost always require additional energy for heating and grinding [13,28,29]. The powder form has various morphologies including euhedral crystals, nanobelts, needle-like and elongated straight crystals, and curly clusters [3,30]. Powders having different morphologies have been synthesized from the pyrolysis of several precursors. They were obtained by the carburizing of the complex esters formed by the condensation reactions between boric acid or its derivatives and polyhydroxylated organic compounds [31–34]. These are polyols [28,35–38], saccharides [39], and polymers [40–43]. Previous studies revealed that the crystalline nature, morphology, particle size distribution, and porosity of the B_4_C vary depending on the consecutive process conditions such as reactants and their molar ratio and the method used as well as pyrolysis temperature and duration. However, the optimum intervals of these variables to obtain the desirable B_4_C powder have never been investigated in detail. Therefore, the objective of the present study was to determine the optimum pyrolysis conditions to obtain the most crystalline B_4_C powder from the precursor of the boric acid/D-mannitol complex ester prepared at a constant molar ratio of 2.

## 2. Materials and methods

### 2.1. Boron carbide synthesis

Boric acid (BA: H_3_BO_3_, 99%) and D-mannitol (MA: C_6_H_14_O_6_, 98%) were supplied by Acros Organics Ltd. (USA) and used without further purification. Because of their increasing solubility with increasing temperature of the aqueous solution of H_3_BO_3_ (3.0 M) and C_6_H_14_O_6_ (3.6 M), both were prepared at 80 °C. The MA solution was slowly added to the BA solution at 80 °C with continuous stirring until it reached a molar ratio of H_3_BO_3_/C_6_H_14_O_6_ = 2 [38]. This homogeneous mixture was then cooled to room temperature with constant stirring and finally left for precipitation for 24 h. The white precipitate obtained at the end of 24 h was a weak acidic complex ester (CE) formed by the condensation reaction between BA and MA. After drying at 100 °C for 4 h, the CE was granulated in an agate mortar. A precursor (PR) sample to synthesize B_4_C was prepared by heating of the granular CE at 400 °C for 4 h, which were determined previously by thermogravimetric analysis. The samples taken from the PR were pyrolyzed under argon flow at various temperatures (1300, 1350, 1400, 1450, and 1500 °C) for 4 h as well as for various durations (1, 2, 3, and 5 h) at 1400 °C.

### 2.2. Instrumental examination

The thermogravimetric and differential thermal analysis (TG/DTA) profiles for the BA, MA, and dried CE were recorded in air between 25 and 500 °C with a heating rate of 10 K/min using a Setaram Labsys apparatus. The X-ray diffraction (XRD) patterns of the BA, MA, PR, and B_4_C powders were collected using a Rigaku D-max 2200 powder diffractometer with CuK_α_ radiation (λ = 0.15418 nm) and a Ni filter. The scanning electron microscopy energy dispersive spectroscopy (SEM-EDS) analysis and SEM images were recorded using a FEI Quanta 400 MK2 instrument. The particle size distribution (PSD) and nitrogen adsorption/desorption isotherms (N_2_-AD) at 77 K were determined for the B_4_C powders synthesized by the pyrolysis. The cumulative and differential PSD curves were recorded using a Malvern Mastersizer 2000 instrument. The N_2_-AD isotherms were obtained using a Quantachrome Instrument (Nova 2200 surface area and pore size analyzer). Prior to each experiment, the starting powder was outgassed under vacuum at 200 °C for 4 h.

## 3. Results and discussion

### 3.1. Thermal analysis of boric acid, mannitol, and complex ester

**Figure 1:**
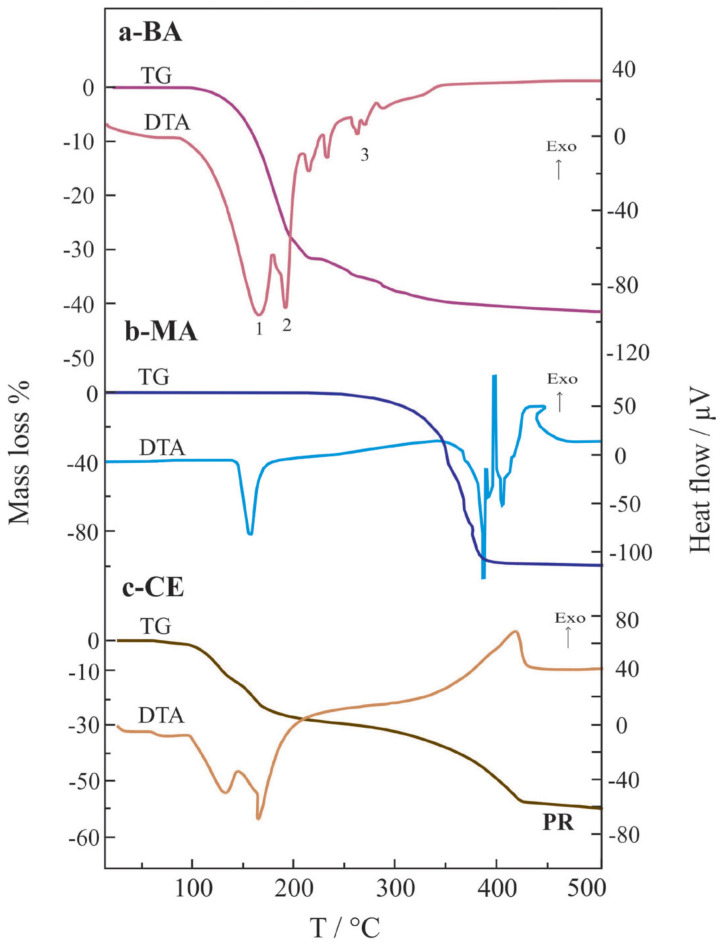
The TG/DTA curves of a) boric acid (BA), b) mannitol (MA), and c) complex ester (CE) containing precursor (PR) position.

Thermogravimetric (TG) curves show the quantitative mass decreases in a material by heating at a constant rate of 10 K/min under air flow. The corresponding differential thermal analysis (DTA) curve represents the endothermic or exothermic nature of the heat exchanges with mass changes. The endothermic decomposition steps of BA to boron oxide (B_2_O_3_) are shown by 1, 2, and 3 in Figure 1a. The endothermic chemical reaction, temperature interval, and mass change percent for each step and total reaction (TR) are respectively given in the following forms:[Table t1-tjc-47-06-1370]

**Table t1-tjc-47-06-1370:** 

1) 6H_3_BO_3_ → 2H_3_B_3_O_6_ + 6H_2_O,	96–163 °C,	27%
2) 2H_3_B_3_O_6_ → H_4_B_6_O_11_ + H_2_O,	163–197 °C,	5%
3) H_4_B_6_O_11_ → 3B_2_O_3_ + 2H_2_O,	197–350 °C,	10%
TR) 2H_3_BO_3_ → B_2_O_3_ + 3H_2_O,	96–350 °C,	42%

Here H_3_B_3_O_6_ and H_4_B_6_O_11_ are the unstable intermediate phases. The temperature intervals and mass changes measured during the chemical reactions and evaluated from TG/DTA curves overlapped with each other, indicating that the endothermic peaks in the DTA curves correspond to the specific reactions.

The endothermic change without mass loss between 160 and 190 °C as seen in Figure 1b is due to the melting of MA. The consecutive endothermic and exothermic changes with the various mass losses between 300 and 400 °C in Figure 1b are due to the tentative thermal decomposition of MA and burning the gaseous organic products, respectively. As a result, MA completely disappears at approximately 400 °C.

Two consecutive endothermic changes with the total mass loss of 30% between 50 and 250 °C in Figure 1c arise from the melting of the CE and thermal decomposition of BA, which is left as an impurity. Furthermore, the exothermic change with 28% mass loss between 250 and 425 °C originates from the combustion of the gaseous products. The carburized CE was used as PR to prepare more crystalline B_4_C powders.

### 3.2. XRD patterns of boric acid, mannitol, and precursor

**Figure 2:**
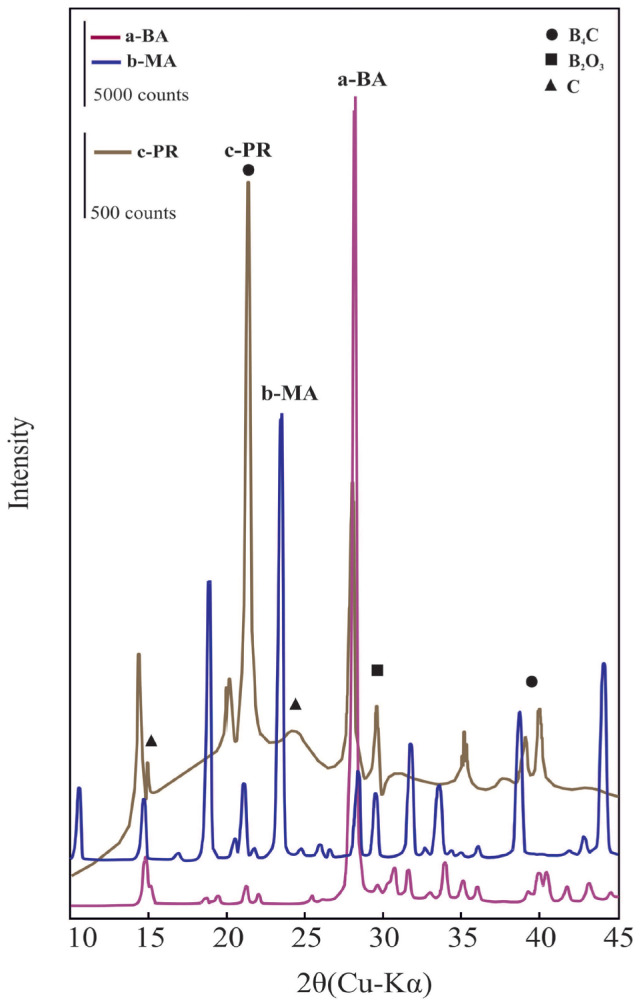
The XRD pattern of a) boric acid (BA), b) mannitol (MA), and c) precursor (PR), which is a heterogeneous mixture of the poor crystalline boron carbide (B_4_C), boron oxide (B_2_O_3_), and elemental carbon (C).

The most intensive XRD reflection for a crystal is a characteristic feature used to distinguish it from others. Accordingly, the characteristic reflections for BA, MA, and PR located at the 2θ positions of 28.0°, 23.5°, and 21.2°, respectively, are seen in Figure 2a, 2b, and 2c. Accordingly, the PR contains a poor crystalline B_4_C and impurities including C and B_2_O_3_.

### 3.3. Optimum pyrolysis conditions of the precursor

**Figure 3:**
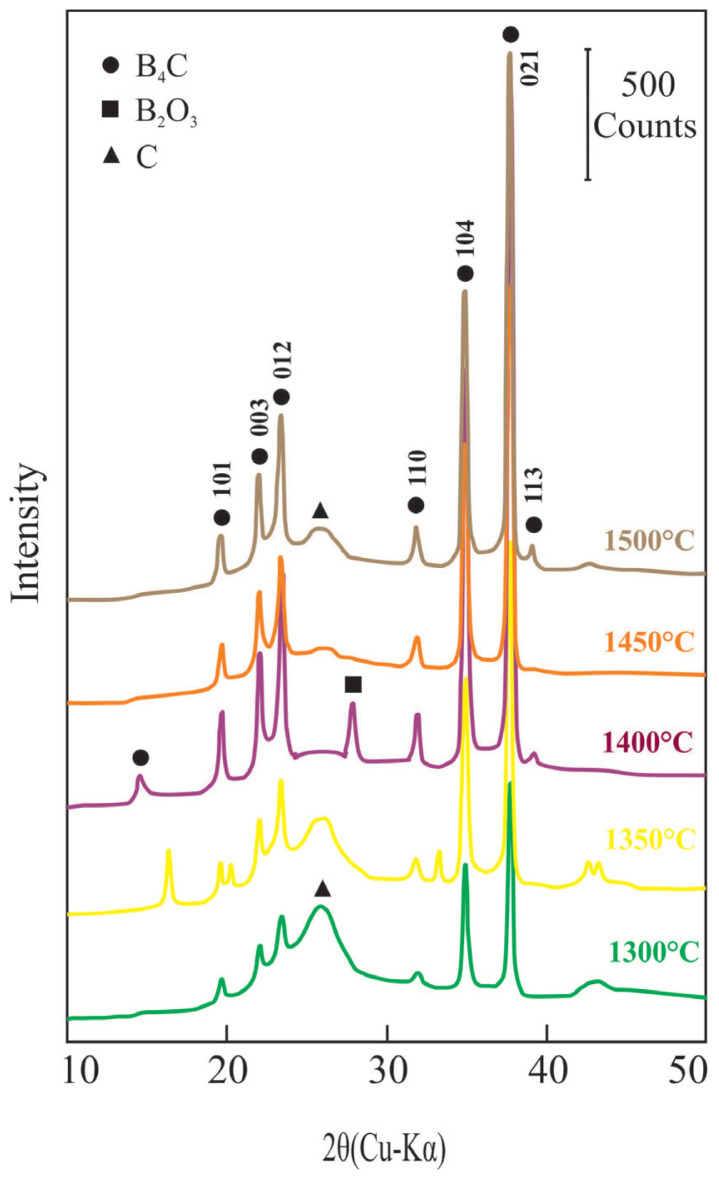
The XRD patterns of the B_4_C powders pyrolyzed for 4 h at different temperatures.

**Figure 4:**
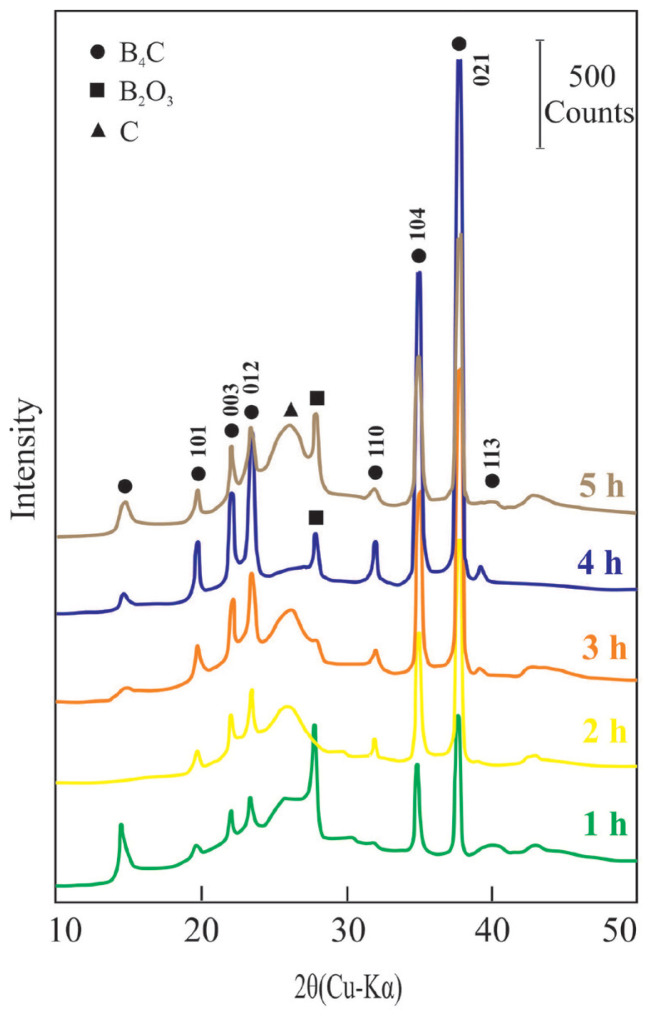
The XRD patterns of the B_4_C powders pyrolyzed at 1400 °C for different times.

In Figures 3 and 4, the XRD patterns show the formation of crystalline B_4_C powders by pyrolysis of the PR at different temperatures for 4 h and for different times at 1400 °C, respectively. The Miller indices (hkl) for the parallel crystal surfaces of B_4_C are given on the corresponding peak intensities. The most intensive XRD reflection for the B_4_C was located at a position of 2θ = 38.7° as seen in Figures 3 and 4 [6,28]. The crystallinity of B_4_C is found to increase at least 15-fold during pyrolysis of the precursor including C and B_2_O_3_ impurities. Although most of the prepared B_4_C powders contain B_2_O_3_ and C as impurities, the one obtained by 4 h pyrolysis at 1400 °C contains only B_2_O_3_, which was consistent with previous studies [37–40]. The present work documents that the existence and amount of elemental carbon vary depending on its pyrolysis temperature and time, as well as the chemical structure of its complex ester and precursor.

**Figure 5:**
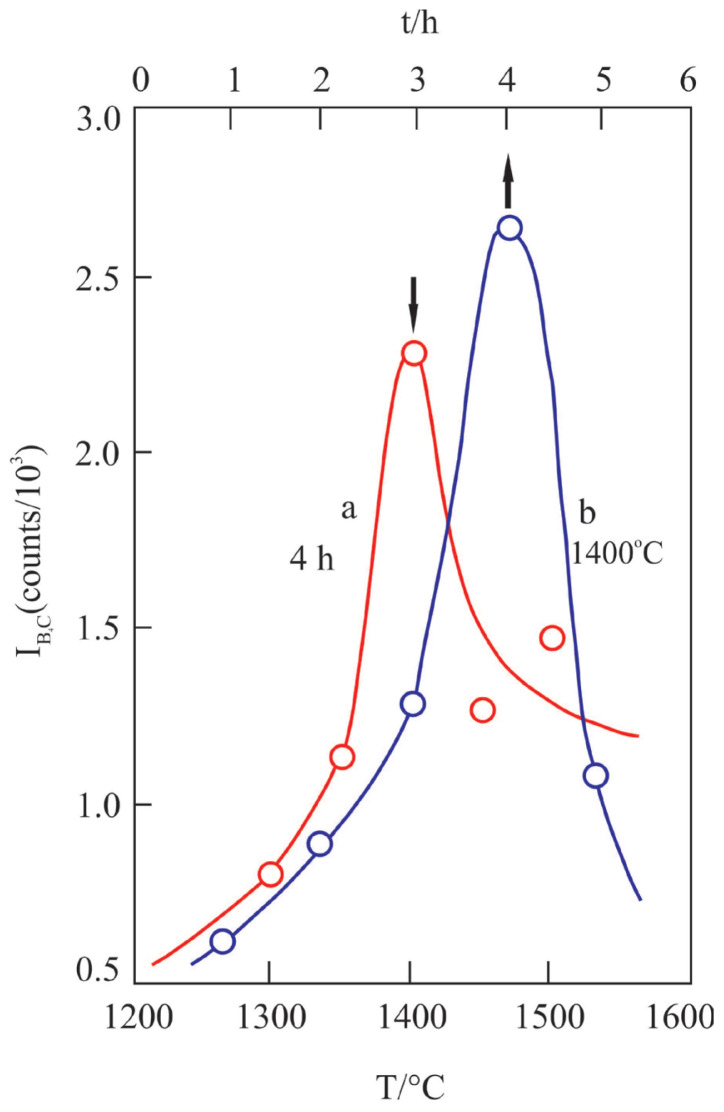
Change in the crystallinity of B_4_C powder depending on a) pyrolysis temperature for 4 h and b) pyrolysis time at 1400 °C.

Intensity changes of the most characteristic reflection (021) of the B_4_C depending on the pyrolysis temperature for 4 h as well as pyrolysis time for 1400 °C are given in Figure 5a and 5b, respectively. The crystalline feature of B_4_C powders after pyrolysis for 4 h changes with temperature, reaching its maximum crystallinity at 1400 °C. Similarly, the crystallinity of B_4_C powders following pyrolysis at 1400 °C changes over time, reaching its maximum crystallinity in 4 h. Accordingly, the optimum pyrolysis temperature and duration for the preparation of the most crystalline B_4_C powders are 1400 °C and 4 h, respectively.

### 3.4. Morphology, particle size, and porosity of the most crystalline B_4_C powder

**Figure 6:**
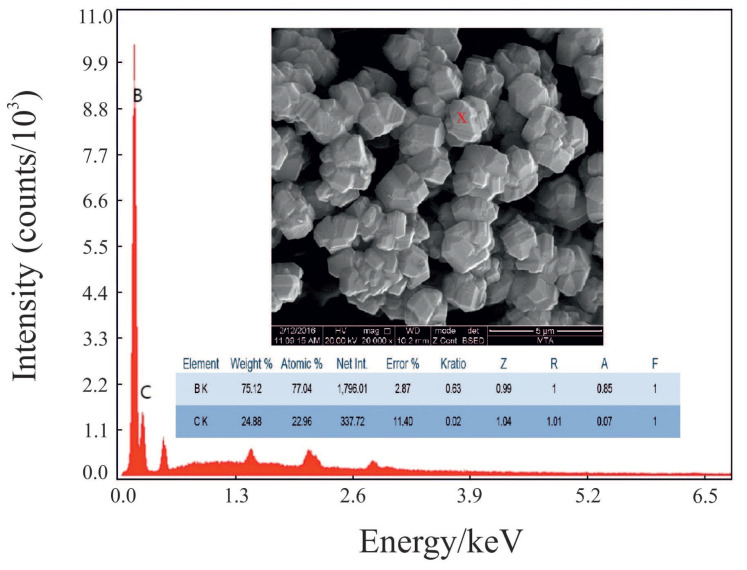
SEM image and spectrum of the B_4_C powder.

**Figure 7:**
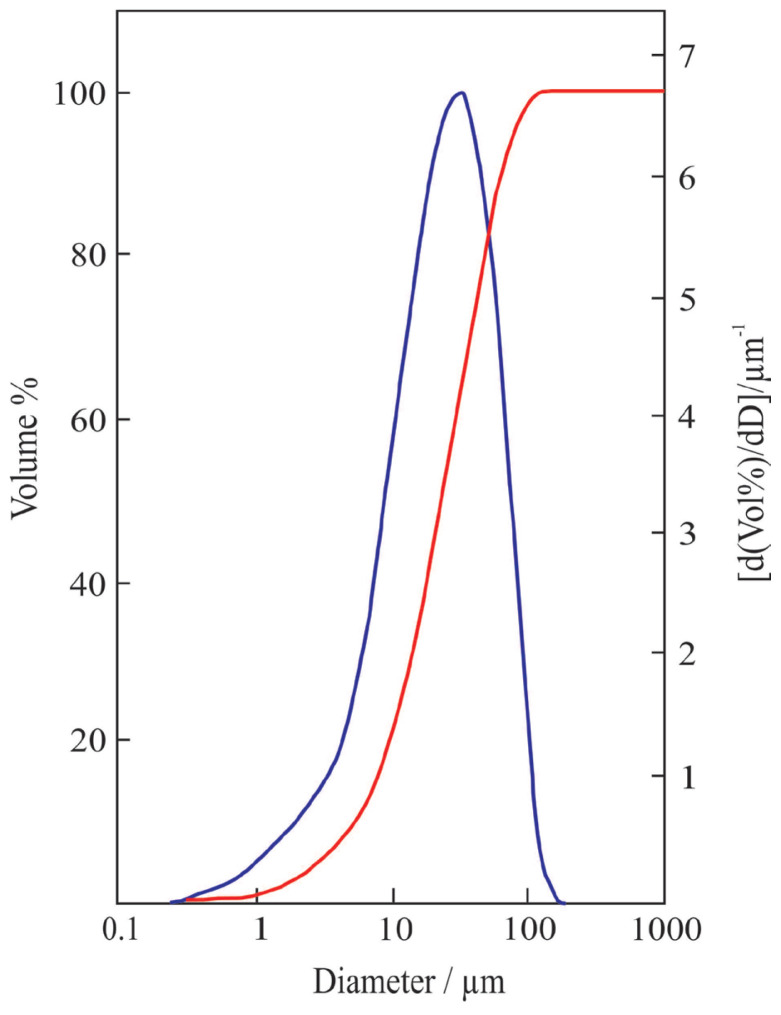
The cumulative and differential particle size distributions of the B_4_C powder.

In Figure 6, the SEM image of the most crystalline powder shows that the microparticles were formed through accumulation of nanocrystals of B_4_C. The cumulative and differential PSD curves indicate that the particle size of the same powder varies between 1 and 100 μm with a mean particle size of approximately 20 μm as seen in Figure 7.

**Figure 8:**
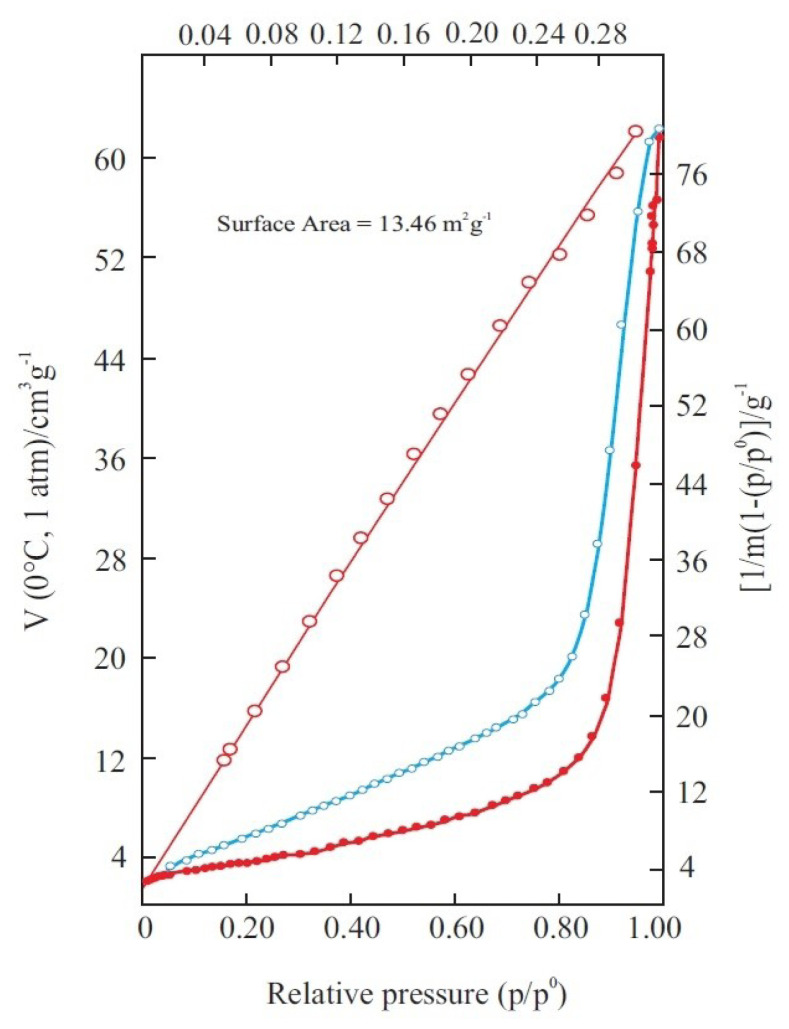
Nitrogen adsorption/desorption isotherms and BET straight line of the B_4_C powder.

The N_2_ adsorption/desorption isotherms of the B_4_C powder determined at −196 °C are given in Figure 8. Here p/p^0^ = x is the relative equilibrium pressure of the adsorption as the ratio of the equilibrium pressure (p) to the vapor pressure (p^0^) of the liquid nitrogen. The adsorption capacity (v/cm^3^ g^−1^) is defined as the volume of adsorbed gas at 0 °C and 1 atm on 1 g of adsorbent solid. Since the adsorption/desorption isotherms are convex to the p/p^0^ axis over the complete range, the B_4_C powder would be considered purely mesoporous adsorbent according to the IUPAC classification [44].

The mesopores filled with the liquid N_2_ by capillary condensation occurred after consecutive monomolecular and multimolecular adsorptions. In contrast, capillary evaporation took place along with the desorption isotherm. Since the capillary condensation begins from the narrowest pores and capillary evaporation from the largest pores a hysteresis loop occurs between the two.

Adsorbed mass on unit mass adsorbate (m/g g^−1^) is defined as gravimetric adsorption capacity. This quantity can be calculated from the volumetric adsorption capacity (v/cm^3^ g^−1^) using the following relationship:


(1) m=(v/V) M,

where V = 22,400 cm^3^ mol^−1^ is the molar volume of gaseous N_2_ at 0 °C and 1 atm, and M = 28 g mol^−1^ is the mol mass of N_2_. Using gravimetric adsorption capacity the linearized Brunauer, Emmett, and Teller (BET) equation [44] derived for multimolecular adsorption can be written in the following from:


(2) $$\frac{1}{m\;(1-p/p^{0})}=\frac{1}{m_{m}c}+\frac{\left(c-1\right)}{m_{m}c}\;\frac{p}{p^{0}},$$

where m_m_ is monomolecular adsorption capacity and c is a constant. The BET straight line is automatically plotted according to this equation as seen in Figure 8. Surface area per unit mass adsorbent is taken as specific surface area (A/m^2^ g^−1^). The A of the B_4_C powder is automatically calculated using the m_m_ value evaluated from the BET plot using the following equation:


(3) $$A=n_{m}N_{A}\sigma_{N_{2}}={\left(m_{m}/M\right)}_{N_{2}}N_{A}\sigma_{N_{2}},$$

where n_m_ (mol g^−1^) = m (g g^−1^)/28 g mol^−1^ is the material content of the monomolecular capacity, N_A_ = 6.02 × 10^23^ mol^−1^ is the Avogadro constant, and S _N_2__ = 6.2 × 10^−20^ m^2^ is the occupied area of the molecule. The A for the B_4_C powder calculated using these relations is A = 13.5 m^2^ g^−1^. This relatively low value also shows that the B_4_C powder has a purely mesoporous structure.

Adsorption and desorption capacities can be also defined as liquid N_2_ volume (V/cm^3^ g^−1^). Their values are calculated using the following relationship:


(4) $$V=\frac{v}{22,400\;{cm}^{3}{mol}^{-1}}V_{N_{2}},$$

where *V_N_*__2__ = 34.65 cm^3^ mol^−1^ is the molar volume of liquid nitrogen at −196 °C. The inner diameter (D) of pores is computed from the corrected Kelvin equation depending on the relative equilibrium pressure of the nitrogen adsorption on a solid.

**Figure 9:**
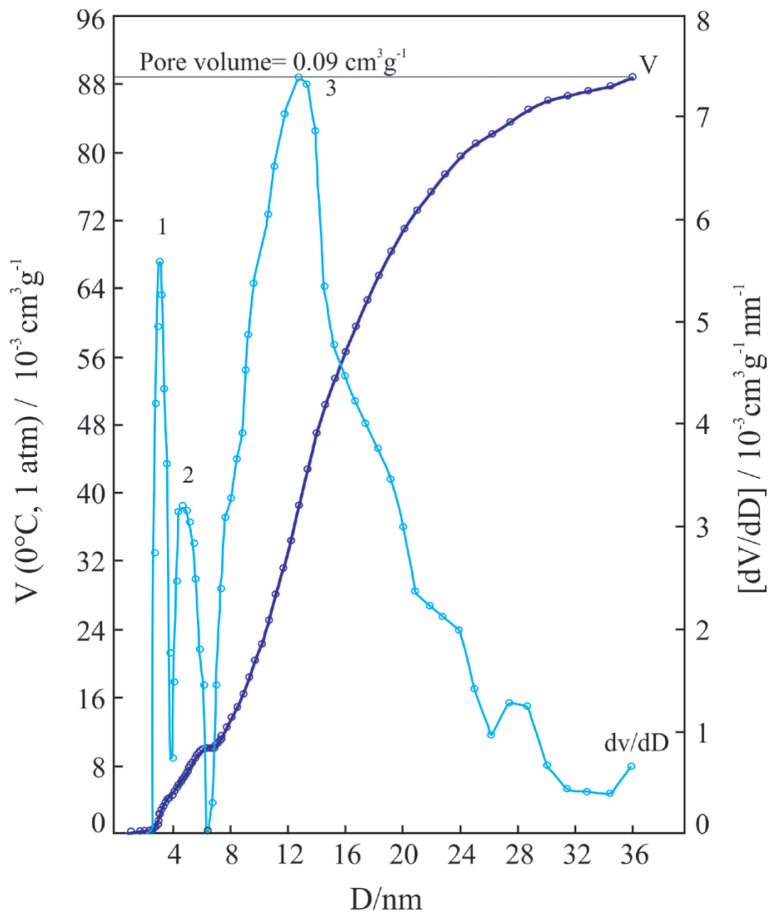
The cumulative and differential pore size distribution of the B_4_C powder.

The cumulative pore size distribution (V-D) and differential pore size distribution (dV/dD-D) are seen in Figure 9. The maximum adsorption capacity as liquid N_2_ volume reading from V-D curves is V = 0.09 cm^3^ g^−1^, which is specific pore volume. This low value shows that the B_4_C powder is a poor mesoporous solid. There are three group mesopores with different volume and range of size as seen in Figure 9. Here the volume and size interval of the groups are respectively 1) 0.004 cm^3^ g^−1^, 3–4 nm; 2) 0.006 cm^3^ g^−1^, 4–7 nm; and 3) 0.080 cm^3^ g^−1^, 7–36 nm. First and second group mesopores would arise from the inner-particle voids, whereas the third group forms the largest mesopores due to intraparticle voids.

## 4. Conclusion

The impurity as well as the crystallinity of B_4_C powders synthesized using the method described in this study vary depending on the temperature and duration of the pyrolysis application. B_2_O_3_ and C or only B_2_O_3_ are formed as impurities. It was determined that the crystallinity of B_4_C powders reached its maximum when they did not contain C as an impurity. This result agrees with the data collected in previous studies. It was observed that adsorption properties such as the specific pore volume and specific surface area of B_4_C powders ranging in size from 1 to 100 μm were low.
